# Flagellate Dermatitis Induced by Shiitake Mushrooms—Clinical Features of a Rare but Characteristic Entity

**DOI:** 10.3390/diagnostics16050692

**Published:** 2026-02-26

**Authors:** Daniel Nette, Patrycja Rogowska, Martyna Sławińska

**Affiliations:** 1 Department of Dermatology, Venereology and Allergology, Faculty of Medicine, Medical University of Gdansk, Smoluchowskiego 17 Street, 80-214 Gdansk, Poland; daniel.nette@gumed.edu.pl (D.N.);; 2 Department of Dermatology, Venereology and Allergology, University Clinical Centre in Gdansk, Smoluchowskiego 17 Street, 80-214 Gdansk, Poland

**Keywords:** flagellate dermatitis, flagellate erythema, shiitake dermatitis, dermatitis, hypersensitivity

## Abstract

We present the case of a 25-year-old male who attended a dermatological online consultation due to a whiplash-shaped, pruritic rash. The lesions in the form of well-demarcated linear erythematous papules, located mainly on the trunk and arms, had first appeared four days prior to the consultation. Chronic disease history was negative and similar symptoms never appeared previously. After reviewing the clinical images and excluding dermatographism, flagellate dermatitis was suspected. The diagnosis was subsequently confirmed when the patient reported having consumed undercooked shiitake mushrooms two days prior to the onset of the lesions. Topical corticosteroids and oral antihistamines were recommended, and the patient was informed about the necessity of high-temperature preparation of shiitake mushrooms in the future. Flagellate dermatitis is a rare entity speculated to be a hypersensitivity reaction to lentinan, a heat-labile polysaccharide found in shiitake mushrooms (*Lentinus edodes*). Symptoms include characteristic papular or vesicular lesions erupting in a linear pattern resembling whiplash marks, usually on the trunk and extremities. While the condition is self-limiting, the awareness of its manifestation is important in order to prevent unnecessary biopsies. Patients should be educated to avoid further exposure to lentinan, as instances of severe reactions following repeated contact have been described.

**Figure 1:**
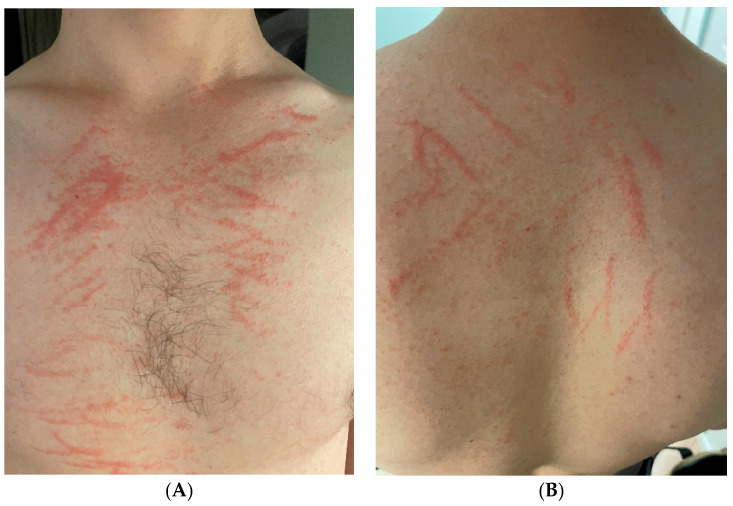
A 25-year-old male patient requested an online dermatological consultation following the eruption of a pruritic rash. The lesions, presenting as erythematous linear papules, were located on the skin of thoracic (**A**), dorsal (**B**), and abdominal regions, as well as the upper limbs, and resembled whiplash marks. The onset had a duration of 4 days at the time of consultation and the lesions persisted despite the patient’s use of oral bilastine (20 mg/day). No provoking factors were suspected by the patient, and he did not recall similar symptoms appearing previously. Chronic disease and medication history were negative. The patient denied repeated mechanical trauma as the possible causal agent for the lesions’ appearance; thus, dermatographia was ruled out. Due to the characteristic clinical picture, flagellate dermatitis was suspected. Further targeted history revealed the ingestion of undercooked shiitake mushrooms by the patient two days before the onset of symptoms, confirming the diagnosis. Symptomatic treatment including emollients, clobetasol ointment 1×/day, and oral bilastine 40 mg/day was recommended, and the patient was reassured regarding the self-limiting nature of this condition. Education was implemented to avoid contact with lentinan in the future by raising the temperature of shiitake mushrooms to at least 145 °C during the preparation of food. Shiitake mushrooms (*Lentinus edodes*), native and frequently present in the cuisine of North-Eastern Asian countries, continuously increase in popularity, having become the second-most cultivated mushroom species worldwide [1]. Apart from their appreciated flavor and nutritional values, they contain numerous bioactive polysaccharides discovered to exhibit antioxidative, anti-inflammatory, anti-aging, antiviral, and antineoplastic properties, and have been used in Asian traditional medicine for centuries [1]. One of the polysaccharides found in shiitake mushrooms is lentinan, speculated to induce a dose-dependent type IV hypersensitivity reaction described as flagellate dermatitis, flagellate erythema, or shiitake dermatitis [2,3,4]. The symptoms arise within 24–72 h after exposure to lentinan and are usually limited to the skin, presenting as characteristic linear, papular, or papulovesicular erythematous lesions resembling “flagellation”, or whiplash marks [2,4] However, instances of other manifestations were noted, with described cases of allergic contact dermatitis [5,6], urticaria [5], photosensitivity [6], mucosal ulcers [7], allergic asthma [8], alveolitis [8], and facial edema with DRESS-like symptoms [2], often following repeated or occupational contact with shiitake mushrooms [2,8]. According to one systematic report, systemic symptoms are experienced by around 5% of the patients and may involve fever, diarrhea, dysphagia, and conjunctivitis, among others [9]. The exact pathomechanism in flagellate dermatitis is unclear. The reported immune-modulating properties of lentinan including the induction of cytokines such as IL-1, IL-2, and TNF-*α* point to a possible Th1 hypersensitivity reaction; specific human leukocyte antigen (HLA) alleles in certain individuals are suggested as another factor [9]. Many studies associate the linear and scratch-like distribution of lesions in flagellate dermatitis with a Koebner phenomenon involving minor trauma during initial periods of intense pruritus, when the serum levels of lentinan are the highest [9,10]. The mild presentations are self-limited and resolve within several days, with possible symptomatic treatment involving topical or oral corticosteroids as well as oral antihistamines suggested in the literature [9]. Patients should be reassured of the self-limiting course of flagellate dermatitis and its typically fast resolution [9]. Moreover, education regarding the avoidance or high-temperature treatment of shiitake mushrooms is important, due to lentinan being a heat-labile compound deactivated above 145 °C [10]. Recommendations should also include avoiding sun exposure, as it has been reported to worsen the symptoms of shiitake dermatitis [11]. In differential diagnosis, chronic inflammatory diseases should be taken into consideration, as flagellate dermatitis was reported as a rare symptom of dermatomyositis or Still’s disease, associated with worse prognosis in the latter condition [12,13]. Cases of flagellate dermatitis as an adverse reaction to chemotherapy were also described, with bleomycin being the most common causative drug [14,15]. In these cases, local minor trauma occurring from scratching is also a suggested mechanism, leading to vascular leakage and localized accumulation of chemotherapeutics [15]. In addition, bleomycin hydrolase expression is limited to subcorneal layers of the epidermis, potentially explaining the increased drug concentration and skin toxicity [15]. Apart from the typical erythematous whiplash-shaped marks, such patients later develop hyperpigmentation within the affected skin, thought to be related to reduced epidermal turnover caused by this chemotherapeutic agent [15]. Other diseases mentioned in differential diagnosis include contact dermatitis and hypereosynophilic syndrome in HIV-positive patients [4,8]. Although shiitake dermatitis is considered to be rare, with around 150 reported cases worldwide [16], the popularization of shiitake mushrooms may lead to the condition becoming more common, facilitating the need for greater awareness of it. Knowledge regarding the characteristic clinical image of flagellate dermatitis potentially allows for an accurate diagnosis even in the settings of teledermatology, thus preventing the use of unnecessary invasive diagnostic procedures.

## Data Availability

The original contributions presented in this study are included in the article. Further inquiries can be directed to the corresponding author.
